# A New Experience

**Published:** 1871-08

**Authors:** H. Scott


### A NEW EXPERIENCE.

BY H. SCOTT.

Mr. R. came to my office to-day for some filling. He is a gentleman of thirty years of age, and of sanguine temperament and robust health. After the filling was completed, he asked my attention to a soreness of the gum between the right central incisor and cuspid of the upper jaw. The lateral had been removed a few years before, on account of an abscess at the root, as the dentist had told him, and the gums had been more or less sensitive and swelled ever since.

The absorption of the alveolus was perfect; but the margins of the gums in the locality was tumefied, extending more or less about the two adjoining teeth. All the mouth, in every other respect, was entirely healthy, the gums being firm, and all the teeth free from calculus. Without a moment's hesitation, I took up a pointed bistoury and made one puncture in the gum, near the incisor, when I was surprised to see jets of pure arterial blood thrown out persistently, and in perfect synchronism with the rythm of the heart. The jets were thrown fully an inch forward, and were so persistent that at least thirty minutes were employed to suppress the hemorrhage, which at last was accomplished by compression. The stream of blood was about equal to a number fifty cotton thread. A curious feature of the case was, that the artery came down from the palatine arch, which I found by compression on the inside, where the pulsations were quite strong. Nearly a pint of blood was lost before I succeeded in arresting its flow. There had been no unusual bleeding when the tooth was extracted.

### AN ABNORMAL CASE.

Miss C., is seventeen years old, and well developed and healthy. She has the permanent central incisors above, well formed, then the permanent cuspids large, and in good order; next to these the deciduous cuspids, sound and firm; and then the bicuspids and molars in natural order. She never had a tooth taken out but the "*loose ones.*"

I have been consulted recently in a case where an upper molar had split open, and I have every reason for believing, by the expansion of the gold with which it was filled. At least three-fourths of the substance of the crown was gold. The walls all round were sufficiently firm, as it seemed to me, to have stood all ordinary uses of the tooth; but they were broken obliquely below the margin of the gum, on the palatine side. The thickness and solidity of the fractured part forbid the conclusion that it could have given way in mastication. The gentleman said that the mallet was used in filling.

